# Supplementary material for: Unexpected Benefits of Multiport Synchrotron Microbeam Radiation Therapy for Brain Tumors
Source: Cancers (Basel). 2021 Feb 24;13(5):936. doi: 10.3390/cancers13050936 (PMC7956531; doi:10.3390/cancers13050936)
Supplement: Supplementary file 1 [file cancers-13-00936-s001.pdf]

Supplementary materials

Unexpected Benefits of Multiport Synchrotron Microbeam Radiation Therapy for Brain Tumors

Laura Eling [1], Audrey Bouchet [1], Alexandre Ocadiz [1], Jean-François Adam [1], Sarvenaz Kershmiri [1], Hélène Elleaume [1], Michael Krisch [2], Camille Verry [3], Jean A. Laissue [4], Jacques Balosso [3,5] and Raphaël Serduc [1,*]

1 INSERM UA7, STROBE, 71 Av. des Martyrs, 38000 Grenoble, France
2 European Synchrotron Radiation Facility, 71, Av. des Martyrs, 38000 Grenoble, France
3 Grenoble University Hospital, 38000 Grenoble, France
4 University of Bern, 3000 Bern, Switzerland
5 CLCC Francois Baclesse, 14000 Caen, France.
* Corresponding author: Raphaël Serduc, INSERM UA07, 71 Av. Des Marthyrs, 38000, Grenoble, 0033476881960 - 0033476882525

Supplementary Methods

Behavioral tests of normal rats

Rats were first tested on three consecutive days for motor deficits. They were placed on a turning cylinder and time, speed and distance until the animal fell off were scored. Second, explorative behavior was assessed on day 4: rats were placed in an open-field arena (ViewPoint LifeSciences, Montreal, Canada) for 20 minutes with a camera installed above the arena. Movements were analyzed using the Viewpoint software for locomotion (time and distance travelled in the whole field (WF) and the center zone) and thigmotaxis ("wall hugging", inversely expressed as entries into the center). Third, the NOR task was performed on day 5. Four hours after animals were habituated to two identical objects, memory function was tested when one of the two objects was replaced with a novel object. During habituation and testing, animals were allowed to move freely within the arena for 10 minutes. Time spent exploring the novel object was calculated as percent of the total time spent exploring both objects. A score >50% indicates more time spent on exploration of the novel object. Statistical analysis was performed with GraphPad Prism® using two sided ANOVA Turkey's multiple comparison test.

Pathology and Immunohistology of brain sections

Vessel morphology was analyzed using Coll-IV (Southern Biotech, 1340-01, 1/500), RECA-1 (Bio-Rad, MCA970R, 1/300) and GLUT-1 (Thermo Fisher, RB 9052-P1, 1/1000). Cell proliferation and DNA damage were visualized using Ki-67 (Thermo Fisher, RM 9106-S1, 1/3000) and Histone H2AX (Merck, 05-636, 1/500), respectively. Macrophage populations were labeled with CD68 (Eurobio, ABC117-6714, 1/1000) and CD11b (Bio-Rad, MCA275R, 1/2000). GFAP (CiteAb, Z0334, 1/500) and NeuN (Merck, MAB377, 1/2000) were used to detect gliosis and neuronal staining, respectively. The oligodendrocyte population was investigated using Olig2 (Abcam, ab109186, 1/2000). Further, other sections were stained using hematoxylin and eosin. Slides were scanned on a ZEISS Axio Scan.Z1 at the Grenoble Institute of Neurosciences (platform "Photonic Imaging Center"); quantitative analysis of vessels and cell populations in the target (tumor or right cortical areas in normal rats) and the contralateral area (left cortical areas) were conducted with the software MoreHisto™. Multiple unpaired t-test analysis (Holm-Sidak correction) was performed with statistical significance set at $p < 0.05$.

Supplementary Results

Multi-port MRT sparsely affected normal rat behavior

Behavioral tests of **r**ats 2, 6 and 12 months after crossed BB irradiation or MRT through 2 or 5 ports were compared with test results of non-irradiated control rats. Inhibition of anxiety-like behavior after MRT, particularly at early time points after MRT5, was revealed by an increased general whole-field exploration and more time spent in the center zone. Another indicator of the "emotional" state of normal rats was given by levels of defecation. Lower levels of defecation found in irradiated animals compared with untreated rats suggest that irradiation did not induce elevated anxiety levels. Additionally, animals were tested for signs of memory loss when they encountered a novel object. No significant differences between irradiated and non-irradiated animals were observed. However, there were moderate deficits in object recognition after MRT5 compared with other groups. Indeed, control animals spent more time exploring the novel object (novel object investigation time ≥50%) than rats exposed to MRT5 during the entire test (novel object investigation <50%). We also tested motor function and coordination of irradiated animals placed on a turning cylinder. Alterations induced after multiport MRT were not significant, but animals irradiated through 5 MRT ports had slightly higher running scores than other rats between 2 and 4 weeks p.i. However, animals with initially increased locomotion after MRT reverted to levels similar to those of control and BB treated rats between 2 and 12 months post exposure.

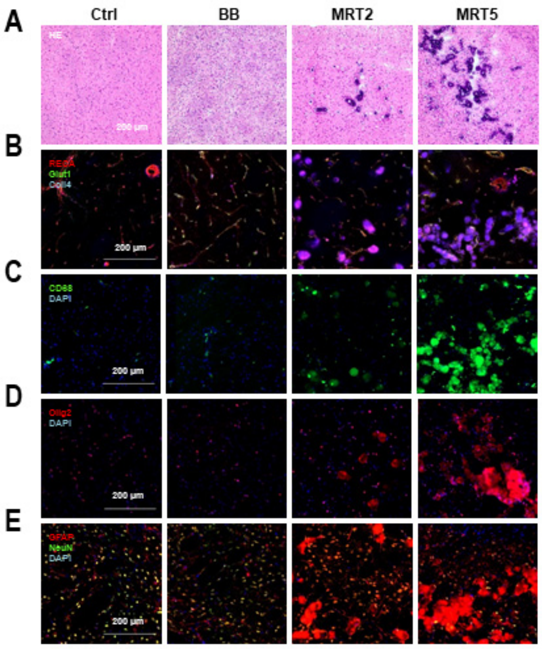

Figure S1. Normal rat brain, center of the irradiation field: Pathology and qualitative immuno-labeling features 12 months after irradiation: Cumulative doses: BB 10 Gy; MRT2 10/752 Gy (valley/peak); MRT5 10/685 Gy (valley/peak). (**A**)Hematoxylin and eosin stain. BB: Not obviously different from the control. MRT2 and MRT5: Microbeam tracks and microcalcifications. (**B**) Collagen-4, RECA-1 and GLUT-1 immuno-staining indicated partial hypo-vascularity in MRT irradiated targets. (**C**) Macrophage infiltration in multiport MRT cross-sections was depicted on CD68-stained images. (**D**) Similarly, microglia density (CD11b-positive cells) increased. (**E**) NeuN immunoreactivity was reduced in the target after MRT irradiation, that of GFAP moderately increased.

Pathology revealed in-target normal tissue damage

Histopathologic changes after BB2 exposure seen on hematoxylin-eosin (HE) stained sections (Figure S1A) were neither substantial nor conclusive. The right frontal cortex of BB animals appeared to be slightly

thinner than that of the left cortex. Neither florid necrotic foci of the gray or white substance, or of blood vessels, nor microcalcifications, astrocytosis or gliosis were seen.

HE-stained sections (Figure S1A) after MRT2 revealed typical microbeam "stripes" corresponding to the hypocellular sites where microplanar beamlet had deposited high peak doses. The microbeam pattern after MRT5 could be partly traced in cortical regions. Microcalcifications, pictured as teardrop-like vacuoles with a basophilic rim, represented the most prominent lesion. They were frequently associated with residual microvascular structures and are well-known signs of vascular radiation damage [1,2]. They were generally located in the frontal cortex, in parts of the orbital cortex and olfactory nuclei, the infralimbic cortex, the caudate nucleus and the thalamus of the right hemisphere. Fibrinoid zones and hemosiderin deposits around small vessels occurred in the MRT beam-crossing area. Right thalamic nuclei, possibly the most vulnerable tissue to microbeam irradiation, presented necrotic areas with granular hemosiderin pigment accumulations measuring around 1 cm in diameter.

In the target regions, immunohistochemistry revealed no consequential changes after BB irradiation regions (Figure S1B–E), compared with untreated brain tissue. In contrast, MRT5, and to a smaller extent MRT2, induced endothelial cell loss and loss of blood vessels (decreased RECA- and increased Glut1-reactivity, respectively, Figure S1B). General reduction of capillaries and an increase in collagen (Coll-reactivity, Figure S1B) were observed where microbeams intersected. Moreover, MRT led to macrophage infiltration around small blood vessels and to increased microglial density in the center of the irradiation field as shown by CD68- and CD11b-immunoreactivities, respectively (Figure S1C,D). We detected moderate fibrillary astrocyte activation in all groups including the control group. GFAP-reactivity in MRT groups was unexpectedly low (Figure S1E). Weaker reactivity to the neuronal marker NeuN (Figure S1E) was found in MRT5 treated animals, probably due to irreversible neuronal cell loss. Microcalcifications in the target of MRT2/5 irradiated animals displayed artefactual reactivity to all immuno-markers.

MRT dose equivalence – Low dose MRT efficiencies *vs.* BB for tumor treatment

Figures S2D, S2E and S2F show tumor volumes measured 7 days after MRT2 or MRT5 (cumulated valley dose of 1, 2, 5 and 10 Gy) and reported on the 9L dose/volume reference curve. Figures S2D and S2E show that MRT2 and MRT5 are more efficient than BB for the control of brain tumor volumes at equivalent valley dose. For instance, MRT5 with a valley dose to the tumor of only 1 Gy (i.e., 5 × 0.2 Gy valley dose per incidence) controlled the tumor as well as BB2 did by deposing 2.4 ± 0.14 Gy. As expected, the more ports applied, the more efficient was MRT. Indeed, for a cumulated valley dose of 10 Gy (MRT2 and MRT5), equivalent biological effects were reached at BB doses of 16.7 ± 4.01 and 20.6 ± 2.76 Gy, respectively (Figure S2G). Curve fits for MRT/BB dose equivalents obtained on T7 (Figure S2E) revealed that the effects of additional MRT ports increased with the prescribed cumulated valley dose. Measured dose equivalences are given in Figure S2F. Reverse analysis of these data provides the valley dose that was required to achieve the same tumor control as the BB dose did. For instance, as shown in Figure 1G, the cumulated valley dose to be delivered for the tumor control brought about by a BB dose of 35 Gy, 7 days after irradiation, was estimated to be equivalent to 17.1 ± 1.09, or 14.6 ± 0.94 Gy when using 2 or 5 MRT ports, respectively.

A

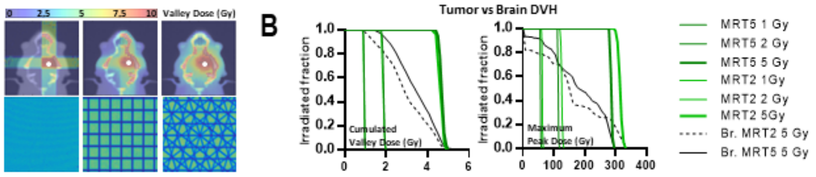

C

Low MRT dose equivalence - Dose prescription and animal group distribution				
	MRT2	MRT5	BB2 ref	Ctrl
Peak (Gy)	2 x 37.6 - 75.2 - 188 - 376	5 x 13.7 - 27.4 - 68.5 - 137		
Valley (Gy)	2 x 0.5 - 1 - 2.5 - 5	5 x 0.2 - 0.4 - 1 - 2		
BB (Gy)			2 x 2 - 5 - 8 - 11 - 17.5	
n MRI/Surv.	15	15	20	4

D

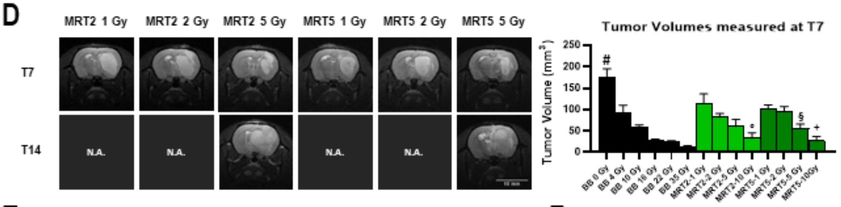

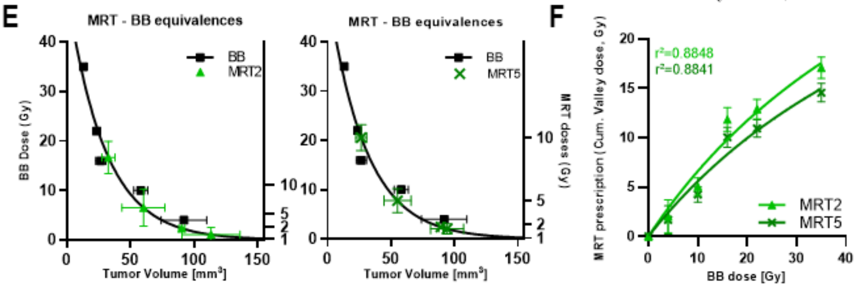

G

Dose (Gy)	Dose Equivalence Cumulated Valley Dose / BB Dose			
MRT Cum. Valley	1	2	5	10
BB eq. (2 MRT ports)	1.1±1.5	2.4±0.2	6.6±3.7	16.7±3.3
BB eq. (5 MRT ports)	2.4±0.1	2.1±1.0	7.8±2.4	20.6±2.6

Dose (Gy)	BB dose anti-tumor efficiency / Equivalent MRT Cumulated Valley Dose to be prescribed				
BB dose	4	10	16	22	35
Cum. Valley (2 MRT ports)	2.0±1.7	5.1±0.9	11.9±1.2	12.9±1.0	17.1±1.1
Cum. Valley (5 MRT ports)	1.7±1.4	4.3±0.8	10.1±1.0	10.9±0.9	14.6±0.9

Figure S2. Improved tumor control of gliosarcomas by increasing numbers of MRT ports and total cumulated valley doses. (**A**) Irradiation geometries and valley dose maps computed in IsoGray for 9L bearing rats. BB was delivered as 2 orthogonal 8 × 8 mm² beams (2 × 2, 5, 8, 11, 17.5 Gy) while MRT (8 × 8mm arrays of 50µm wide microbeams, spaced 400 µm) was delivered as 2 orthogonal (2 × 0.5, 1, 2.5, 5 Gy cumulated valley dose) or 5 arrays spaced by 36° intersecting in the right caudate nucleus (5 × 0.2, 0.4, 1, 2 Gy cumulated valley dose). (**B**) Whole brain and tumor dose-volume histograms computed for BB and 2 and 5 MRT ports for different MRT cumulated valley doses (1, 2, 5 Gy). Valley doses and peak doses are plotted as the cumulated dose and the maximum (cumulated intersecting microbeam doses) deposited in a 1 × 1 × 1 mm³ CT voxel. (**C**) Irradiation parameters and group size (n) for animal follow up (MRI and survival). (**D**) Representative T_2 weighted MR images acquired on 9L bearing rats on day 7 and 14 after MRT irradiation through 2 and 5 ports (1, 2 and 5 Gy). 9L tumor volumes measured on MR images 7 days after irradiation for BB (4, 10, 16, 22 and 35 Gy, reference 9L tumor response to conventional irradiation) and MRT irradiations through 2 or 5 ports (1, 2, 5 and 10 Gy). N.A : not applicable. (**E**) MRT / BB equivalence doses: MR tumor volumes obtained at day 7 after irradiation (T7) were forced on the fitted reference 9L tumor response curve (black line) for MRT 2 ports (left) and 5 ports (right) and the total cumulated valley doses were reported on the right x-axis. (**F**) Relationship between the total cumulated MRT (2 and 5 ports) valley dose prescribed that is equal to the BB dose delivering equivalent tumor control efficiency. (**G**) Dose equivalence between total cumulated valley dose (for 2 and 5

ports MRT) and BB: a 10 Gy valley dose delivered to the tumor via 2 or 5 MRT ports will have an effect of 16.7 Gy and 20.6 Gy BB, respectively. Total cumulated valley dose to be delivered to reach BB dose tumor control efficiency: to reach an effect of 35 Gy BB on 9L tumors, a 17.1 or a 14.6 Gy cumulated valley dose has to be delivered by 2 or by 5 MRT ports, respectively. In each panel, BB group: solid black; MRT 2 ports: light green; MRT 5 ports: dark green. Data are plotted as mean +/− SEM. Significance was determined using unpaired t-tests for $p < 0.05$, and noted as *0Gy vs. all treatment groups except MRT2-1Gy, °MRT2-10Gy vs. MRT2-1/2Gy, §MRT5-5Gy vs. MRT5-1/2Gy, +MRT5-10Gy vs. MRT5-1/2/5Gy.

SI References

1. Shanley, D.J. Mineralizing microangiopathy: CT and MRI. *Neuroradiology* **1995**, *37*, 331–333, doi:10.1007/BF00588350.
2. Harwood-Nash, D.C.; Reilly, B.J. Calcification of the basal ganglia following radiation therapy. *Am. J. Roentgenol. Radium Ther. Nucl. Med.* **1970**, *108*, 392–395, doi:10.2214/ajr.108.2.392.
